# Ethyl loflazepate as a treatment for patients with idiopathic and psychogenic taste disorder

**DOI:** 10.1186/s13030-022-00246-1

**Published:** 2022-08-08

**Authors:** Ken-ichiro Sakata, Hiroyuki Hato, Jun Sato, Takashi Iori, Noritaka Ohga, Haruhisa Watanabe, Yutaka Yamazaki, Yoshimasa Kitagawa

**Affiliations:** 1 Department of Oral Diagnosis and Medicine, Division of Oral Pathobiological Science, Faculty of Dental Medicine and Graduate School of Dental Medicine, Hokkaido University, Kita-13 Nishi-7, Kita-ku, Sapporo, Hokkaido 060-8586 Japan; 2 Department of Gerodontology, Division of Oral Health Science, Faculty of Dental Medicine and Graduate School of Dental Medicine, Hokkaido University, Kita-13 Nishi-7, Kita-ku, Sapporo, Hokkaido 060-8586 Japan

**Keywords:** Ethyl loflazepate, Idiopathic taste disorder, Psychogenic taste disorder

## Abstract

**Background:**

Ethyl loflazepate (EL) is a benzodiazepine derivative that has been reported to activate the gustatory cortex. Our department routinely uses EL as a first-line treatment for idiopathic and psychogenic taste disorders, although little has been reported in the literature with respect to patient outcomes, so we conducted a retrospective study examining its safety and efficacy.

**Methods:**

Between 2008 and 2020, 49 patients (14 males and 35 females; mean age, 62.1 years) were diagnosed with taste disorders and received EL as their only treatment for > 14 days. Severity of taste disorder was evaluated using the paper disc method by Sakai et al., and treatment efficacy was evaluated using the Visual Analog Scale, wherein patients gave subjective ratings for their symptoms (reductions by > 50% after administration of EL for 4 weeks were defined as improvements).

**Results:**

Results showed that the improvement rates for patients with idiopathic and psychogenic taste disorders were 55 and 70%, respectively. Additionally, the majority (78%) improved within 2 weeks, and side effects were mild (seven cases with drowsiness and one case with dizziness).

**Conclusions:**

We conclude that EL is an appropriate first-line medication for patients with idiopathic and psychogenic taste disorders.

## Background

Taste disorders have become a hot topic worldwide as a sequela of COVID-19. In developed countries, the number of patients with taste disorders is expected to increase due to population aging. Taste disorders are more than just a sensory abnormality; it is a serious problem that greatly reduces quality of life. When left untreaded for a long period, taste disorders can lead to malnourishment due to loss of appetite and eventually promotes the frailty cycle that progresses from weight loss to sarcopenia.

Ethyl loflazepate (EL) is a benzodiazepine derivative that is safe for elderly individuals and is well documented to treat anxiety and dental psychosomatic disorders [1–3]. Note worthily within the last two decades, research has also reported that this drug activates the gustatory cortex [4]. In particular, Takahashi et al. [1] reported that EL can successfully correct or improve abnormal taste function emerging spontaneously. Our department systematically diagnoses and treats taste disorders [5], and we frequently use EL as a first-line therapy when they are suspected of arising spontaneously or having a psychological origin. At present, few investigations have evaluated outcomes following the administration of EL in patients with idiopathic or psychogenic taste disorders [6], so we conducted a retrospective study examining its safety and efficacy among cases presented to our team.

## Methods

### Participants

**Fig. 1:**
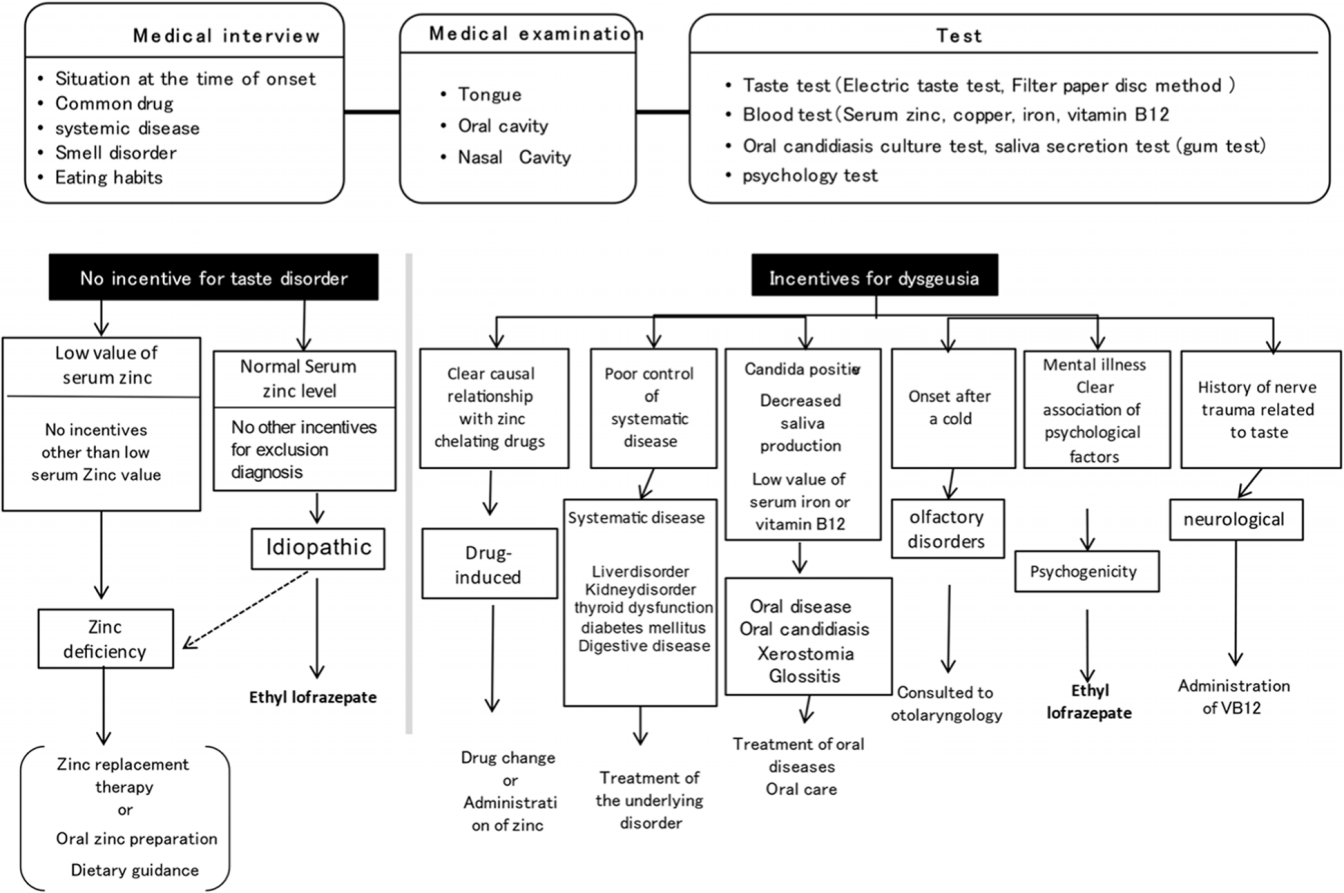
Diagnosis and treatment of taste disorders

Between January 2008 and January 2019, patients complaining of gustatory dysfunction were admitted to the Department of Oral Medicine at Hokkaido University Hospital Dental Center and treated with single EL. In total, the safety of EL was assessed in 55 patients, although 6 dropped out early because of side effects. This rendered a final number of 49 patients [14 males and 35 females; age, 62.1 years (range, 29 × 84 years); and average duration of the disease, 25 months] for efficacy analysis, given that they received the drug for > 2 weeks (Fig. 1 and Table 1). The target patients were diagnosed only with idiopathic or psychogenic taste disorder based on the criteria listed in Fig. 1.

**Table 1:** Taste disorders are caused by several factors

• **Zinc deficiency**: Serum zinc level is less than 70 μg/dl and there are no other obvious causes.	
• **Idiopathic:** There are no other obvious causes of taste disorder, excluding psychogenicity.	
• **Oral disease:** Those with taste disorders that are closely related to oral diseases, such as candidiasis, xerostomia, glossitis, and tongue coating	
• **Psychogenic:**Psychological factors clearly related to the onset and progress of the symptoms.	
• **Systematic disease:** Currently, the state of control of systemic disease is poor (liver disorder, renal disorder, diabetes, and gastrointestinal disorder).	
• **Drug induced:** Described in the information regarding side effects, and has a clear causal relationship.	
• **Post common cold:** Awareness of taste disorders following a common cold.	
• **Olfactory disorders:** Simultaneous taste and olfactory disorder; taste is normal and only olfaction disorder is present.	
• **Iatrogenic:**Those that developed after middle ear surgery or laryngeal microsurgery and palatine tonsil surgery.	

With regard to efficacy analysis, EL was a first-line therapy for 42 patients and a second-line therapy for 7 patients who were previously treated for oral candida with the antifungal drug Floried gel®, yet they experienced no improvements in taste. Upon examination, 22 and 27 patients were diagnosed as having an idiopathic or psychogenic taste disorder, respectively, per the criteria listed in Fig. 1. Of note, the former has no established etiology (Table 1), whereas the latter is considered to have a psychological basis according to various tests (e.g., CMI, SDS, and PHQ-9). Subjective symptoms of taste disorders were judged according to the definition shown in Table 2 [5]. A blood test and bacterial culture test of the tongue were performed on all cases to differentiate the cause of taste disorders.

**Table 2:** Subjective symptoms of taste disorders

Phantogeusia: Having a certain taste although there is nothing in the mouth.	
Hypogeusia: The taste of food fades.	
Taste hypersensitivity: The taste of food becomes stronger.	
Different taste: Feeling a different taste from the original.	
Dissociable: A specific taste cannot be felt.	
Bad taste: A bad taste in the mouth regardless of the flavor of the food.	

This study was conducted with the approval of the Hokkaido University Hospital Independent Clinical Research Review Committee (Approval No. 018–0381: Clinical study in patients with taste disorders.). All eligible patients provided informed consent for inclusion before participating in the study. The study was conducted in accordance with the Declaration of Helsinki.

### Clinical examination

Severity of taste disorder was evaluated using Taste Discs® (Sanwa Kagaku, Nagoya) and the method outlined by Sakai et al. [7]. Scores for perception of sweetness, saltiness, sourness, and bitterness in the tympanolar region that were < 3.5, 3.5–4.5, 4.5–5.5, and > 5.5 were recognized as normal taste function, mild taste disorder, moderate taste disorder, and severe taste disorder, respectively. Measurement of serum zinc levels was performed using an atomic fluorescence analyzer (AA240FS, Agilent, Santa Clara, CA, USA), with normal values ranging from 70 to 111 μg/dL according to Harrison’s Principles of Internal Medicine [8].

### Administration of EL

One tablet (1 mg) of EL was administered before going to bed at night. If a patient experienced daytime sleepiness or heart flutters, the dosage was reduced to 0.5 mg.

### Evaluation of efficacy

Treatment efficacy of EL was evaluated using the Visual Analog Scale (VAS) [9]. Patients who exhibited improvements after 4 weeks were defined as those with > 50% decrease in subjective symptoms, and those regarded as unchanged had < 50% decrease in subjective symptoms. Two or more people, including the responsible author and other authors, performed the evaluation.

In two cases, no improvement in patients’ conditions was detected after 2 weeks, so administration of EL was discontinued, and patients were given another agent for duration of 2–4 weeks. Consequently, these individuals were only evaluated at the time when they decided to discontinue EL treatment.

### Statistical analysis

Improvement rates according to age, gender, duration of the disease, subjective symptoms, cause of taste disorders, period of EL administration, length of time to experience an effect from EL, serum zinc values, and severity of taste disorder were compared using Fisher’s exact test, and *p* < 0.05 was considered significant. The type and incidence of adverse events were examined as a means to determine safety.

## Results

**Fig. 2:**
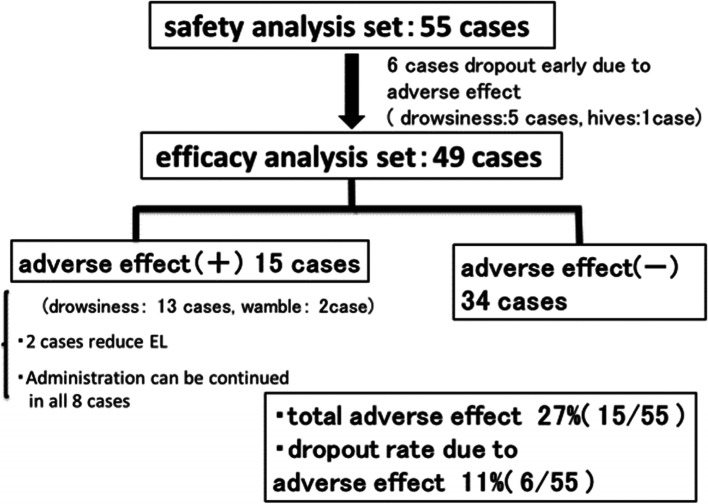
Profile of this study

Among the 55 patients assessed for treatment safety, 6 dropped out because of drowsiness and 1 dropped out because of urticaria. Shortly after discontinuing EL administration, these adverse effects lessened or abated in the five cases (Fig. 2).

Out of the 49 patients evaluated for treatment efficacy, phantogeusia was observed in 26 cases, hypogeusia in 10 cases, and complaints of different tastes in 7 cases. In addition, 31 patients showed an improvement in taste disorder (i.e., rate of 63%), 18 remained unchanged (i.e., rate of 37%), and no patients did not exhibit worse symptoms. Moreover, no significant differences in improvement rates for the indices delineated above were observed (Table 3). Adverse events of drowsiness or dizziness were reported by seven patients and one patient, respectively, although all symptoms were minor, so administration of EL continued. For two patients, side effects from EL were slightly more intense, so the drug’s dosage was reduced to 0.5 mg.

**Table 3:**
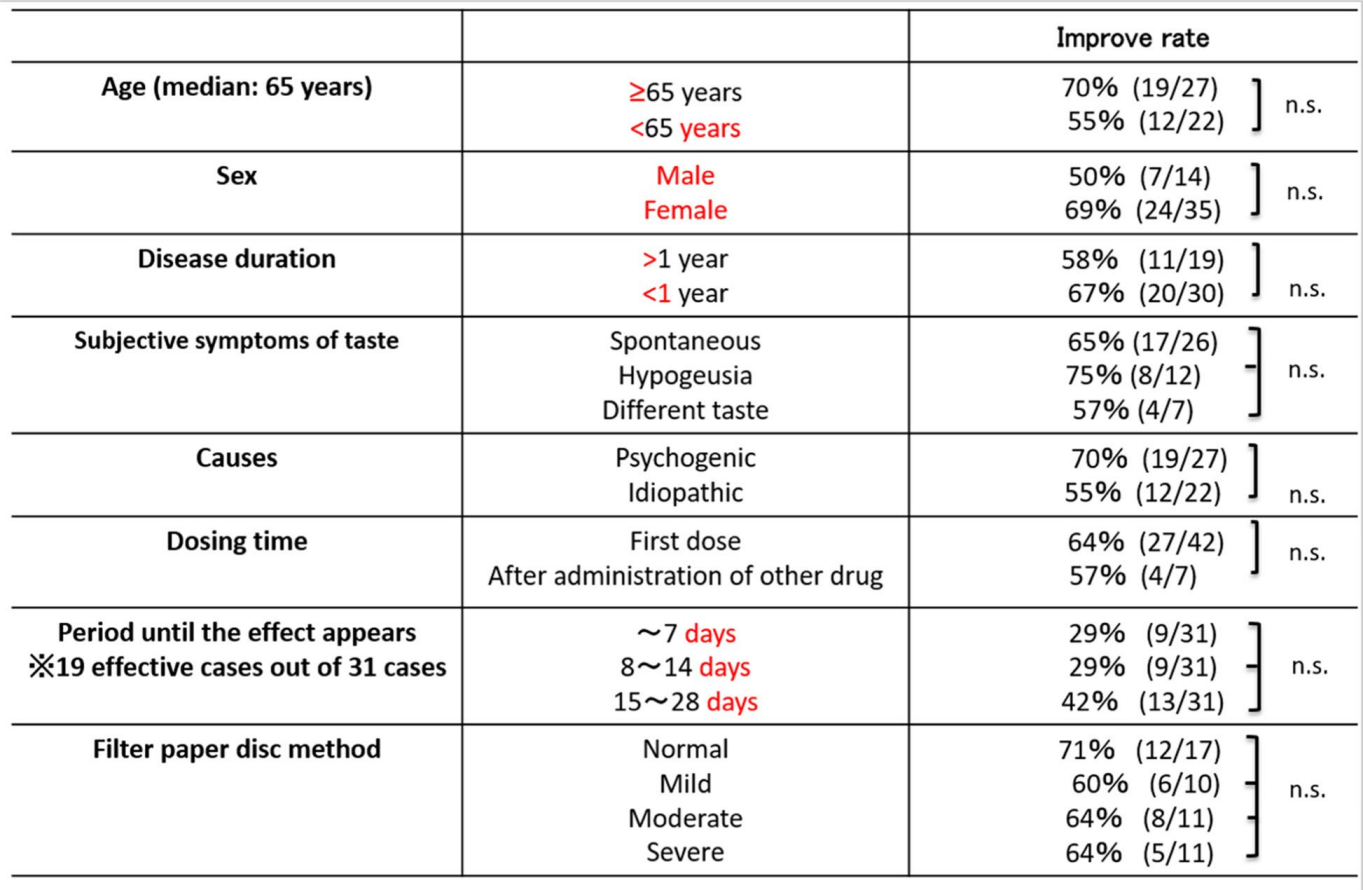
Results for effectiveness analysis

Across all patients who regained at least some taste function, they were able to cease treatment after maintaining a consistent dosage of EL over the course of 2–3 months. One patient relapsed 8 months after the end of the administration.

## Discussion

Causes of taste disorders are multitudinous and can be attributed to the common cold, zinc deficiency, olfactory dysfunction, and systemic, oral, iatrogenic, idiopathic, and psychogenic diseases [10]. In our department, the subjective symptom classification of taste disorder is classified as described in Table 2, whereas the cause was classified as described in Table 1. Conventionally, most of the subjective symptoms of taste disorder include hypogeusia, with other studies reporting a few occurrences of phantogeusia, different taste, and dissociable taste disorders, which are types of taste disorders. Our department has reported that phantogeusia accounts for around 40% of the subjective symptoms, followed by hypogeusia [5]. Phantogeusia has been treated as a psychogenic disease, given its specific complaint emphasizes psychogenic involvement. However, the rates of oral candidiasis at the Department of Oral Medicine, Hokkaido University Hospital has been quite high [5]. Similarly, this report administered an antifungal drug as the initial drug to seven cases. Patients who visit the dentistry department undergo examination for oral candidiasis to determine the cause of taste disorders. In the future, as the social structure becomes more complex and the population ages, we expect an increase in the number of patients coming in for complaints of subjective abnormal taste due to two factors, psychogenic and candida. Therefore, it is necessary to consider oral disease as a possible cause without being bound by psychogenic prejudice for cases complaining of constant bitterness or saltiness despite nothing in the oral cavity. Moreover, there are cases in which it is difficult to diagnose and identify the cause due to various complaints from patients. The reason is that there are cases where taste disorder is suspected to be caused by multiple predispositions. Moreover, given that the symptoms of taste disorders are not cause-specific, the therapeutic effect cannot be evaluated unless confirmed by a taste test or similar methods. Considering the numerous difficult cases, our department systematically treats taste disorders, as shown in Fig. 1. Treatment for the majority of these issues is well established, but effective options for managing idiopathic and psychogenic taste disorders remain to be unidentified [5]. Within the realm of otolaryngology, zinc replacement therapy has traditionally been recommended for patients with idiopathic and psychogenic taste disorders [11–13], which is why our department has actively administered polaprezinc. Unfortunately, improvement rates have not exceeded 22%.

With this background, one contemporary investigation by Takahashi et al. [1] reported that EL was successful at correcting or improving abnormal taste functions emerging spontaneously, whereas another study by Shimura et al. [4] found that EL specifically stimulated the gustatory cortex. Moreover, unlike other benzodiazepine drugs and polaprezinc [14–17], EL has been known to act rapidly and last for quite a long time (i.e., half-life of 122 h), prevents or treats anxiety, and appears to have minimal side effects (e.g., less memory loss and drowsiness) [18, 19]. Furthermore, El is safe for older individuals and has been well documented for treating anxiety and dental psychosomatic disorders [1–3]. Thus, we believed that EL would be an ideal therapy for the cases presented to our hospital.

In this study, there was an overall improvement rate in taste disorders of 63% (31/49): 55% (12/22) for patients with idiopathic taste disorders and 70% (19/27 for patients with psychogenic taste disorders. In addition, 78% (12/31) of those for whom EL was effective experienced improvement within 2 weeks of administration. Surprisingly, no significant differences in improvement rates by age, gender, duration of the disease, subjective symptoms, cause of taste disorders, period of EL administration, length of time to experience an effect from EL, serum zinc values, or severity of taste disorder were observed. Prior research, in particular, has demonstrated that upon treatment, the duration of a disease decreases significantly after ≥6 months and it takes a while for patients to experience improvements [11], yet for our cohort, there was no significant difference in improvement rates between patients with a course of > 1 year vs. < 1 year. This finding bears witness to the expediency of EL over other medications commonly employed in such instances.

Concerning lack of improvement or side effects, the incidence rate was 27% (15/49). Five patients chose to discontinue EL treatment in favor of different drugs because they experienced no alteration in their condition, and the rest predominantly had mild sleepiness. Written on the medical packaging for EL are the main side effects, including psychiatric disorders (e.g., drowsiness and reduced cognition and concentration) and central and peripheral nervous system disorders (e.g., stabilization and speech problems, and head sensations), and a minor side effect, that is, loss of taste. Importantly for the latter, the frequency of loss of taste is described as < 0.1%, but we could not locate evidence of this phenomenon in the literature nor did anyone in our cohort complain of this. A mere amount of 1 mg of EL was administered to our patients because the drug’s manufacturers state that the daily dosage for insomnia is 2 mg. Hence, this would likely induce strong sleepiness. Going forward, healthcare professionals should be diligent in informing their patients regarding the aforementioned side effects and monitoring them carefully after EL administration. If the effect of EL on taste disorders was observed, maintenance therapy for approximately 3 months was performed, and the oral administration was discontinued following EL is gradually reduced. Our study limitation of the current study is the small number of cases and taste test using Taste Discs® after EL administration has not been performed. We plan to accumulate more cases in the future and consider undertaking a prospective study.

## Conclusions

This study examined the safety and efficacy of EL in patients with idiopathic and psychogenic taste disorders between 2008 and 2020 and found that it resulted in an overall improvement rate of 63% (31/49). In particular, among the 24 patients for whom EL was effective, 78% (24/31) experienced improvements within 2 weeks and an improvement rate of 50% was observed when the disease had lasted a year or longer. In addition, adverse reactions occurred in 13 patients, with the most common being drowsiness. We conclude that EL monotherapy could be given as a first-line treatment for patients with idiopathic and psychogenic taste disorders.

## Data Availability

We are not able to share our data since sharing data is not permitted by our hospital or the ethics committee.

## Abbreviation

EL: Ethyl loflazepate
